# Supplementary material for: From scales to armor: Scale losses and trunk bony plate gains in ray‐finned fishes
Source: Evol Lett. 2021 Mar 23;5(3):240–50. doi: 10.1002/evl3.219 (PMC8190451; doi:10.1002/evl3.219)
Supplement: Supplementary file 1 — Fig. S1 Reconstruction through stochastic mapping of the scale / scaleless trait evolutionary history on a 304 species phylogeny presented by Hughes et al (2018). Fig. S2 Reconstruction of the scale / scaleless trait evolutionary history on a 304 species phylogeny presented by Hughes et al (2018). Fig. S3 Reconstruction of the scale / scaleless trait evolutionary history, accounting for phylogenetic uncertainty, on the 304 species phylogeny presented by Hughes et al (2018). Fig. S4 Reconstruction through stochastic mapping of the scale / scaleless trait evolutionary history on the 11,638 species phylogeny presented by Rabosky et al (2018). Fig. S5 Reconstruction of the scale / scaleless trait evolutionary history on the 11638 species phylogeny presented by Rabosky et al (2018). Fig. S6 Reconstruction through stochastic mapping of the presence/absence of trunk plates trait evolutionary history on the 304 species phylogeny presented by Hughes et al. (2018). Fig. S7 Reconstruction of the presence/absence of trunk bony plates (TBP) evolutionary history on the 304 species phylogeny presented by Hughes et al (2018). Fig. S8 Reconstruction of the presence/absence of trunk bony plates (TBP) evolutionary history, accounting for phylogenetic uncertainty, on the 304 species phylogeny presented by Hughes et al (2018). Fig S9. Reconstruction through stochastic mapping of the presence/absence of trunk bony plates (TBP) evolutionary history on the 11638 species phylogeny presented by Rabosky et al (2018). Fig. S10 Reconstruction of the the presence/absence of trunk bony plates (TBP) evolutionary history on the 11,638 species phylogeny presented by Rabosky et al (2018). Fig S.11 Ancestral state reconstruction of the presence / absence of scales in actinopterygians indicates that scale reacquisition is rare and should be considered with caution. Table S1. List of 304 species of Actinopterygii and their classification for three characters. Table S2 List of 11,638 species of Actinopte [file EVL3-5-240-s001.docx]

SUPPLEMENTARY MATERIAL

Title: ***From scales to armour: scale losses and trunk bony plate gains in ray-finned fishes***

Authors: Alexandre Lemopoulos, Juan I. Montoya-Burgos

**Fig. S1** Reconstruction through stochastic mapping of the scale / scaleless trait evolutionary history on a 304 species phylogeny presented by Hughes et al (2018). Blue color corresponds to the scaleless phenotype while red color corresponds to the presence of scales. Probability of the ancestral trait state at each node are based upon 5000 tree simulations.
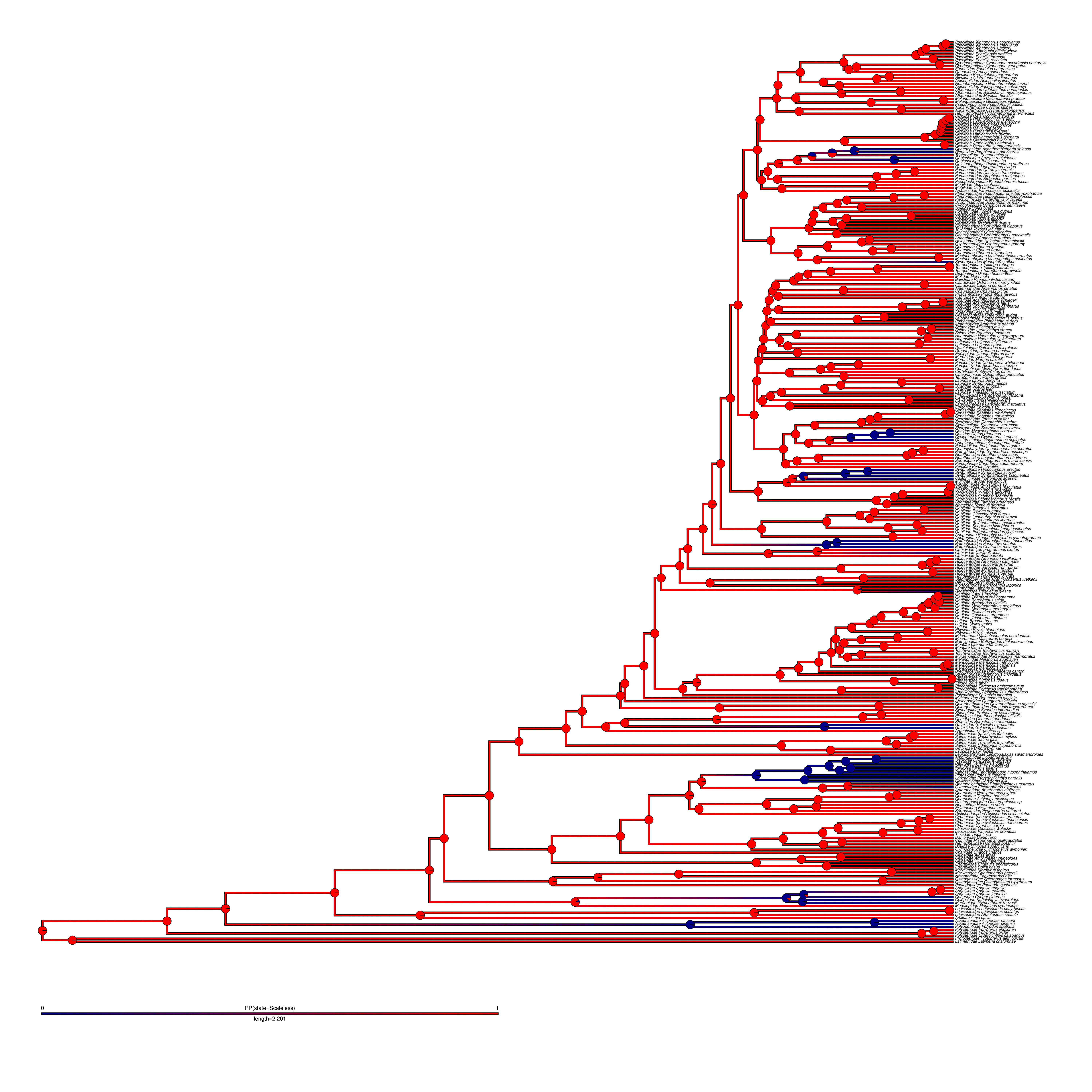


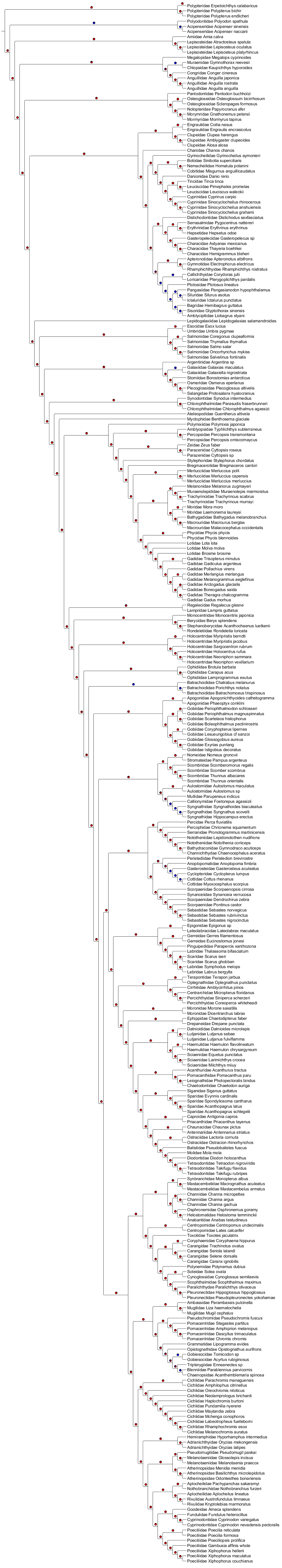
 **Fig. S2** Reconstruction of the scale / scaleless trait evolutionary history on a 304 species phylogeny presented by Hughes et al (2018). The analysis was performed on 50 million iterations on Bayestrait 2.0 (Pagel et al. 2004) using the AddMRCA method. Blue color corresponds to the scaleless phenotype while red color corresponds to the presence of scales.

**Fig. S3** Reconstruction of the scale / scaleless trait evolutionary history, accounting for phylogenetic uncertainty, on the 304 species phylogeny presented by Hughes et al (2018). The analysis was performed on 50 million iterations on Bayestrait 2.0 (Pagel et al. 2004) using the AddMRCA method. A set of 1000 trees was used to account for phylogenetic uncertainty (see material and methods in main text). Blue color = scaleless phenotype; red color = presence of scales.


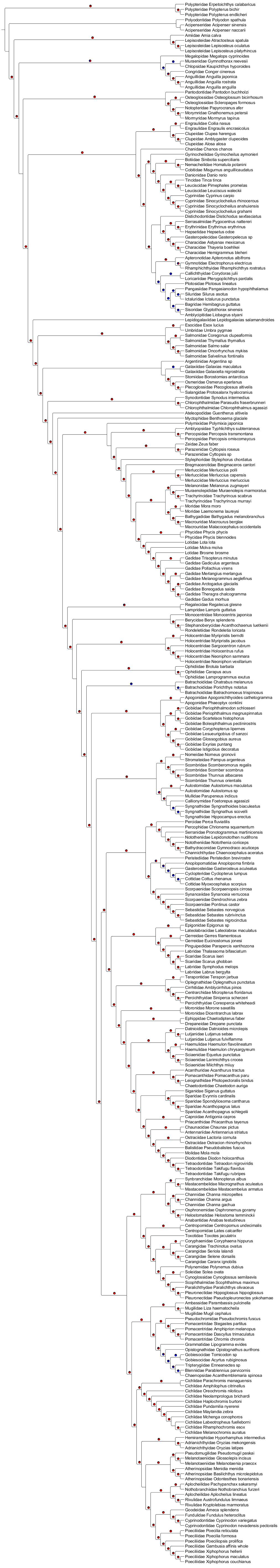


**Fig. S4** Reconstruction through stochastic mapping of the scale / scaleless trait evolutionary history on the 11,638 species phylogeny presented by Rabosky et al (2018). Blue color corresponds to the scaleless phenotype while red color corresponds to the presence of scales. Probability of the ancestral trait state at each node are based upon 250 tree simulations.

**
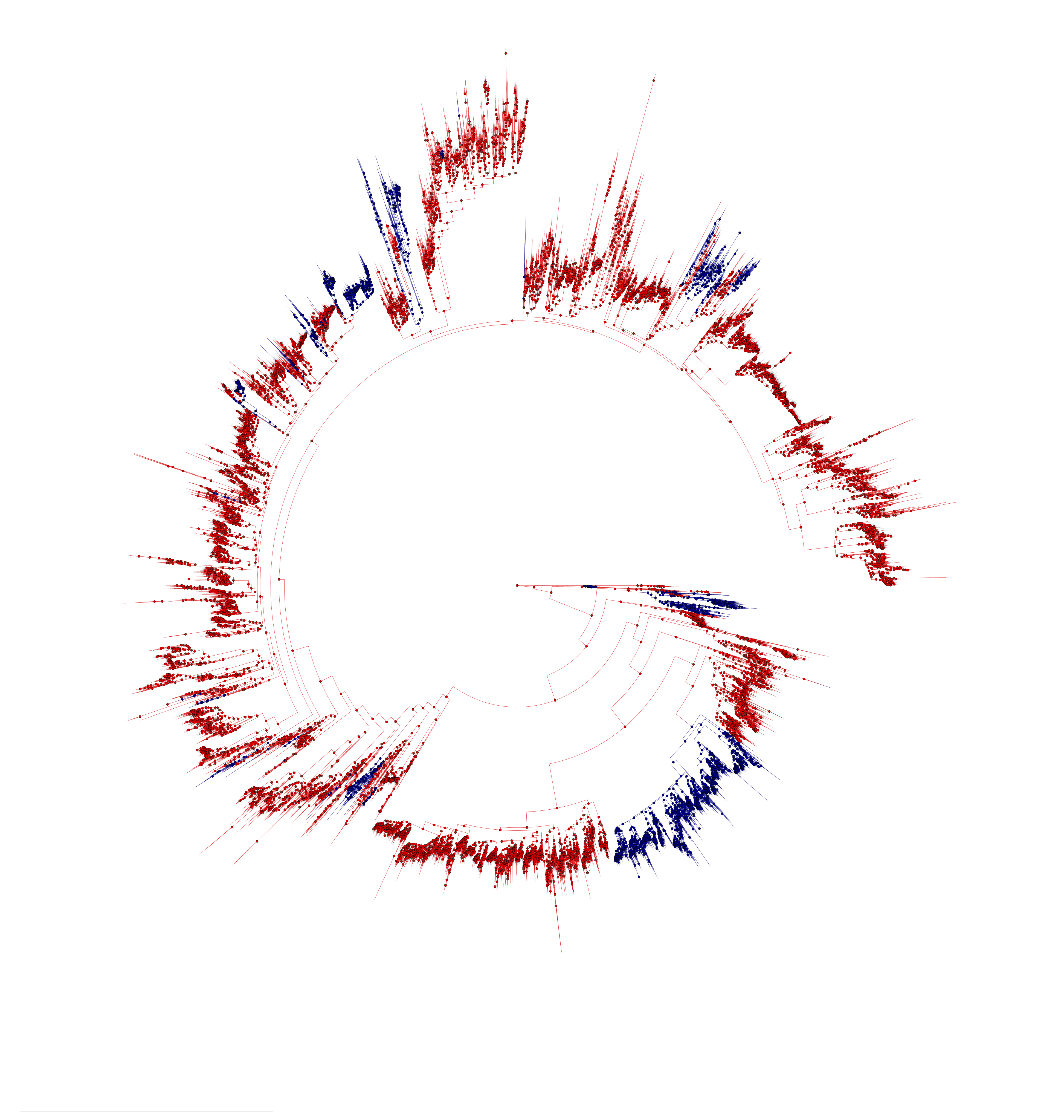
**

**Fig. S5** Reconstruction of the scale / scaleless trait evolutionary history on the 11638 species phylogeny presented by Rabosky et al (2018). The analysis was performed on 10 million iterations on Bayestrait 2.0 (Pagel et al. 2004) using the AddMRCA method. Blue color corresponds to the scaleless phenotype while red color corresponds to the presence of scales.

*File attached (due to a large size)*

**Fig. S6** Reconstruction through stochastic mapping of the presence/absence of trunk plates trait evolutionary history on the 304 species phylogeny presented by Hughes et al. (2018). Red color corresponds to the presence of plates while blue color corresponds to the absence of plates. Probability of the ancestral trait state at each node are based upon 5000 tree simulations.


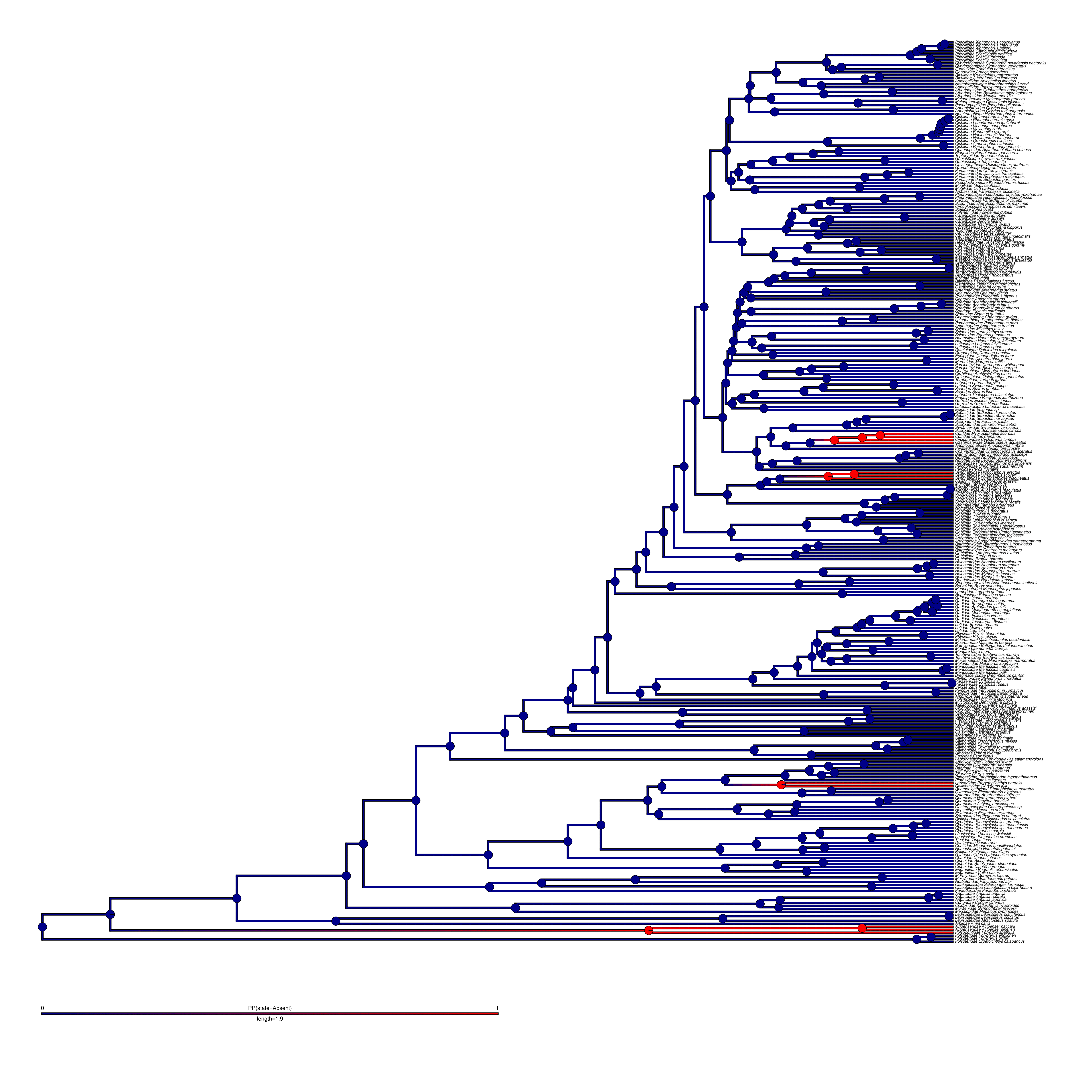


**
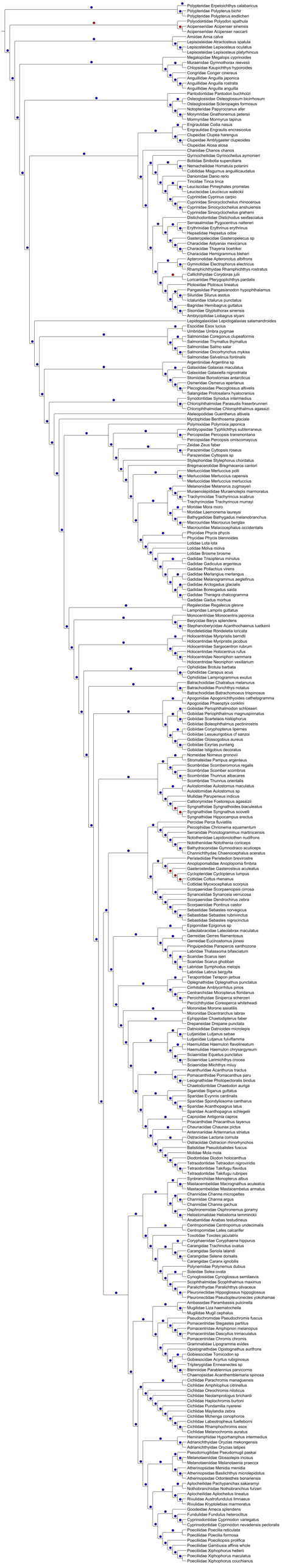
Fig. S7** Reconstruction of the presence/absence of trunk bony plates (TBP) evolutionary history on the 304 species phylogeny presented by Hughes et al (2018). The analysis was performed on 50 million iterations on Bayestrait 2.0 (Pagel et al. 2004) using the AddMRCA method. Blue color corresponds to the absence of plates while red color corresponds to the presence of plates.


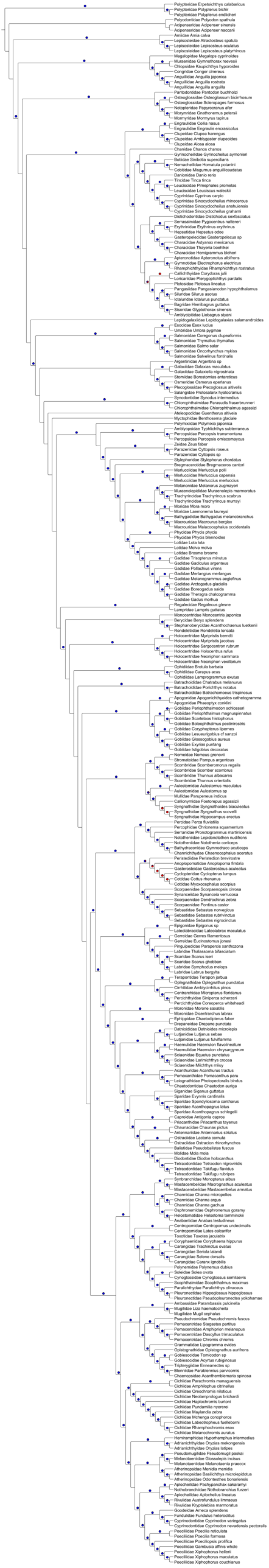
 **Fig. S8** Reconstruction of the presence/absence of trunk bony plates (TBP) evolutionary history, accounting for phylogenetic uncertainty, on the 304 species phylogeny presented by Hughes et al (2018). The analysis was performed on 50 million iterations on Bayestrait 2.0 (Pagel et al. 2004) using the AddMRCA method. A set of 1000 trees was used to account for phylogenetic uncertainty (see material and methods in main text). Blue color corresponds to the absence of TBP while red color corresponds to the presence of TBP.

**Fig S9.** Reconstruction through stochastic mapping of the presence/absence of trunk bony plates (TBP) evolutionary history on the 11638 species phylogeny presented by Rabosky et al (2018). Red color corresponds to the presence of TBP while the blue color corresponds to the absence of TBP. Probability of the ancestral trait state at each node are based upon 250 tree simulations.


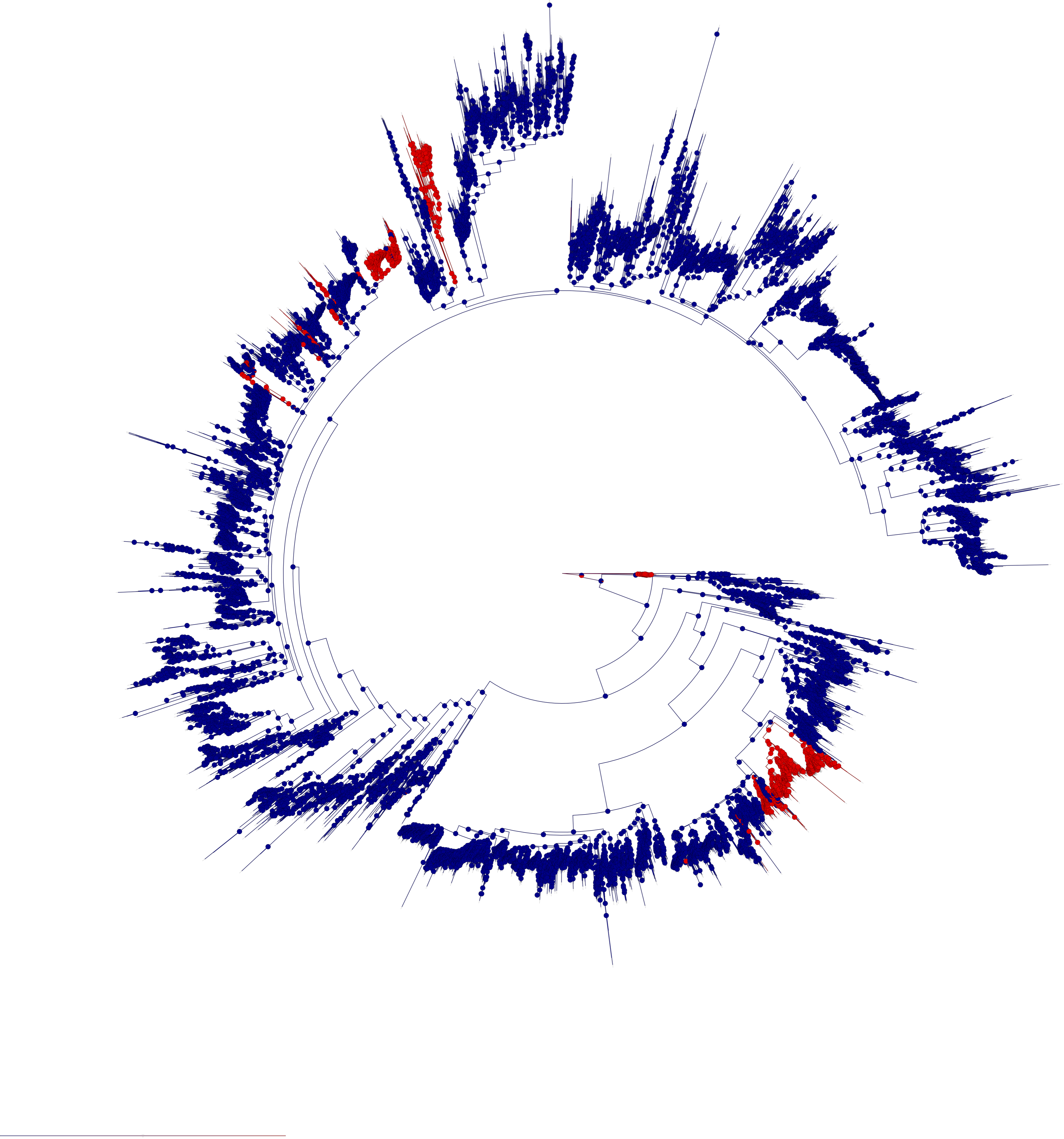


**Fig. S10** Reconstruction of the the presence/absence of trunk bony plates (TBP) evolutionary history on the 11,638 species phylogeny presented by Rabosky et al (2018). The analysis was performed on 10 million iterations on Bayestrait 2.0 (Pagel et al. 2004) using the AddMRCA method. Blue color corresponds to the absence of TBP while red color corresponds to the presence of TBP.

*File attached (due to a large size)*

**Fig S.11** Ancestral state reconstruction of the presence / absence of scales in actinopterygians indicates that scale reacquisition is rare and should be considered with caution. The reconstruction was performed using Bayestrait 2.0 on the dataset of 11,638 species published by Rabosky *et al*. (2018). Scale reacquisition is globally infrequent and, in addition, some cases might be doubtful as reacquisition events were reconstructed within groups for which phylogenies are poorly resolved and/or contradictory with published group-specific phylogenies (in cases A, and C to K), while another is potentially due to an artifactual reconstruction (case B). Hereafter, subtrees with the ancestral states are presented for every case of scale reacquisition (A to K). Blue color corresponds to the absence of scales while red color corresponds to the presence of scales.

1. A scale reacquisition was inferred in the *Anguilla* genus. However, the taxonomic position of the *Anguilla* genus is still uncertain with regards to other published phylogenies (Johnson *et al*. 2012; Santini *et al*. 2013).
2. A scale reacquisition is inferred in the Mastacembelidae family (Synbranchiformes) when analysing the phylogeny of Rabosky *et al.* (2018). This scale reacquisition can be explained by the successive branching of two scaleless lineages (*Indostomus* +*Monopterus* and *Chaudhuria*) before the divergence of the scaled lineage comprising *Macrognathis* + *Mastacembelus*. Yet, the Synbranchiformes group has been previously described as paraphyletic (Kawahara *et al.*, 2008), suggesting unclear phylogenetic relationships that may lead to misleading reconstructions. Based on the 304 species phylogeny of Hughes *et al*. (2018), the stochastic mapping reconstruction (cf. Fig S1) did not show a scale reacquisition event in this lineage because the reduced taxonomic sampling did not include more than one scaleless species.
3. A scale reacquisition was identified in the *Clariger* / *Luciogobius* genera. However, in this reconstruction, the *Luciogobius* genus is polyphyletic, in contrast to the group-specific phylogeny of the Luciogobiidae (Yamada *et al*. 2009)
4. The phylogenetic position of the genus *Parapeneus* and related genera does not correspond to the phylogeny of Smith *et al*. (2014). Consequently, the scale acquisition could be the result of a wrong topology. Further investigation is needed to clarify this case.
5. A scale reacquisition has been inferred in a lineage of Notothenioidei. It has also been reconstructed using stochastic mapping (see Fig.S4). However, the phylogeny of this lineage is different than the one presented in Near *et al.* (2018) and this lack of phylogenetic resolution may cause problems in the reconstruction.
6. In the *Lycodes* genus, the phylogeny we used (Rabosky *et al.* 2018) does not correspond with the one presented in Turanov *et al*. (2017). Consequently, the reconstructed scale reacquisition event must be taken with caution.
7. In *Cryptacanthodes*, the phylogeny of Rabosky *et al.* (2018) does not correspond with the one in Radchenko et al. (2011). The lack of a robust phylogenetic resolution may question the scale reacquisition event.
8. In the phylogeny we used (Rabosky *et al.* 2018), the clade comprising *Ocosia*, *Neosebastes* and related genera has a topology which is very different from the topology presented in Smith *et a*l. (2018). This discrepancy reveals the weakness of the phylogenetic resolution in this clade, which may question the secondary scale acquisition inferred in *Ocosia* and *Neosebastes.*
9. The scale acquisition event in the genus *Lophiocharon* may be due to its misplacement nested within the *Histriophryne* genus and thus breaks its monophyly, in contradiction with the phylogenetic hypothesis of Arnold & Pietsch (2012).
10. The scale acquisition in *Perulibatrachus* / *Halobatrachus* is potentially due to a lack of phylogenetic resolution as the phylogeny of Rabosky *et al.* (2018) contradicts with the one proposed by Rice & Bass (2009).
11. The *Stomias* genus is polyphyletic in the phylogeny we used (Rabosky *et al.* 2018) and this is in contradiction with the phyloegny of Kenaley *et al.* (2014), which may explain this secondary acquisition of scales.


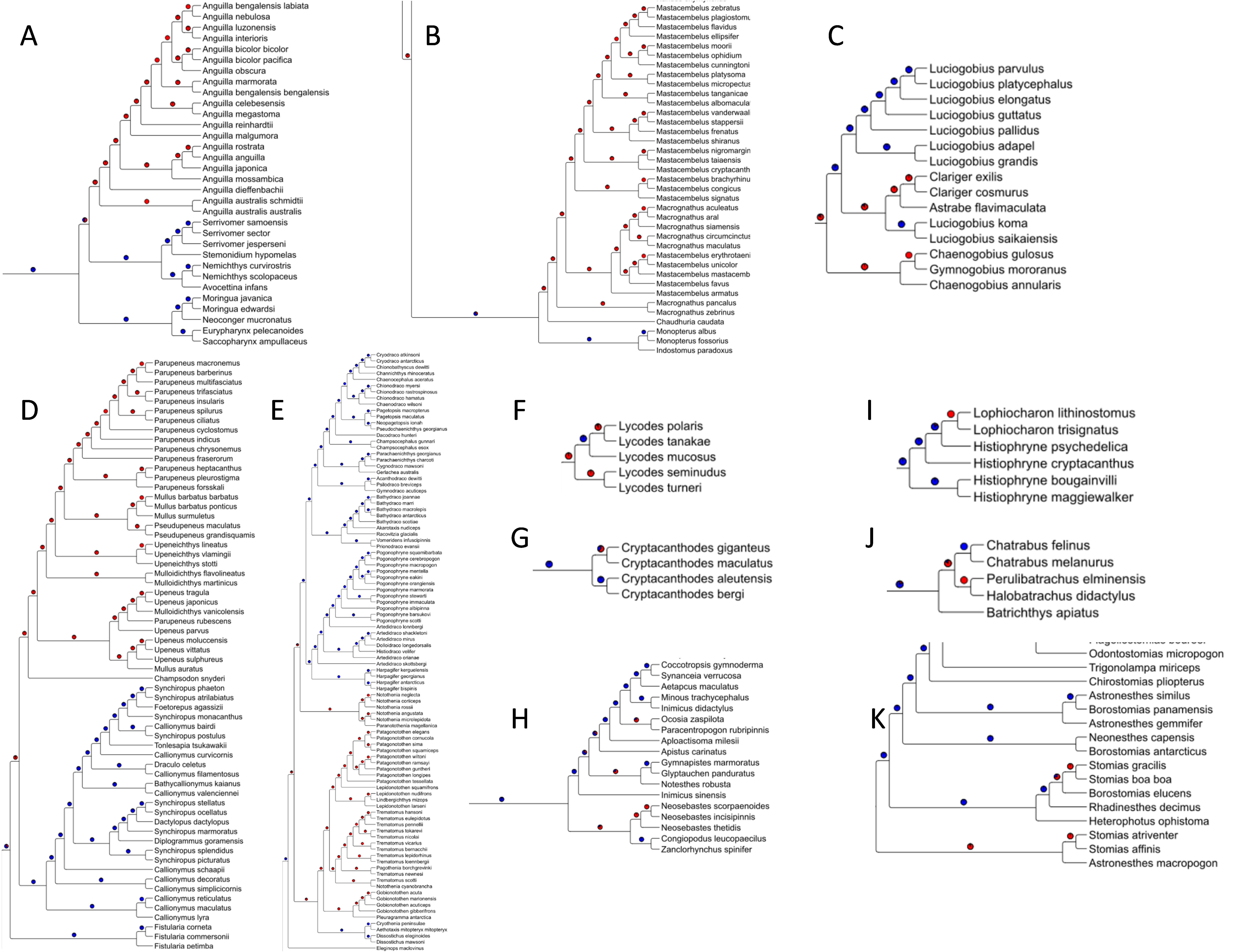


**Table S1.** List of 304 species of Actinopterygii and their classification for three characters. Presence (1) or absence (0) of scales, of trunk bony plates (TBP), and finally the habitat preference (0 = open water, 1 = benthic). The information about the traits was collected in two books (Moyle and Cech 2004; Nelson et al. 2016) and in Fishbase (Froese and Pauly 2011). When information was lacking or unclear in these three main sources, species characteristics were extracted from the specialized literature, given in the column "Reference".

| Species | Scales | Plates | Habitat pref. | Reference |
| --- | --- | --- | --- | --- |
| Latimeriidae_Latimeria_chalumnae | 1 | 0 | 0 |  |
| Protopteridae_Protopterus_aethiopicus | 1 | 0 | 1 |  |
| Polypteridae_Erpetoichthys_calabaricus | 1 | 0 | 1 |  |
| Polypteridae_Polypterus_bichir | 1 | 0 | 1 |  |
| Polypteridae_Polypterus_endlicheri | 1 | 0 | 1 |  |
| Polyodontidae_Polyodon_spathula | 0 | 1 | 1 | Weisel et al. 1975 |
| Acipenseridae_Acipenser_sinensis | 0 | 1 | 1 | Hilton et al. 2011 |
| Acipenseridae_Acipenser_naccarii | 0 | 1 | 1 |  |
| Amiidae_Amia_calva | 1 | 0 | 0 |  |
| Lepisosteidae_Atractosteus_spatula | 1 | 0 | 0 |  |
| Lepisosteidae_Lepisosteus_oculatus | 1 | 0 | 0 |  |
| Lepisosteidae_Lepisosteus_platyrhincus | 1 | 0 | 0 |  |
| Megalopidae_Megalops_cyprinoides | 1 | 0 | 0 |  |
| Muraenidae_Gymnothorax_reevesii | 0 | 0 | 1 | Böhlke et al. 1989 |
| Chlopsidae_Kaupichthys_hyporoides | 0 | 0 | 1 |  |
| Congridae_Conger_cinereus | 0 | 0 | 1 |  |
| Anguillidae_Anguilla_japonica | 1 | 0 | 1 |  |
| Anguillidae_Anguilla_rostrata | 1 | 0 | 1 |  |
| Anguillidae_Anguilla_anguilla | 1 | 0 | 1 |  |
| Pantodontidae_Pantodon_buchholzi | 1 | 0 | 0 |  |
| Osteoglossidae_Osteoglossum_bicirrhosum | 1 | 0 | 0 |  |
| Osteoglossidae_Scleropages_formosus | 1 | 0 | 0 |  |
| Notopteridae_Papyrocranus_afer | 1 | 0 | 0 |  |
| Morymridae_Gnathonemus_petersii | 1 | 0 | 0 |  |
| Mormyridae_Mormyrus_tapirus | 1 | 0 | 0 |  |
| Engraulidae_Coilia_nasus | 1 | 0 | 0 |  |
| Engraulidae_Engraulis_encrasicolus | 1 | 0 | 0 |  |
| Clupeidae_Clupea_harengus | 1 | 0 | 0 |  |
| Clupeidae_Amblygaster_clupeoides | 1 | 0 | 0 |  |
| Clupeidae_Alosa_alosa | 1 | 0 | 0 |  |
| Chanidae_Chanos_chanos | 1 | 0 | 0 |  |
| Gyrinocheilidae_Gyrinocheilus_aymonieri | 1 | 0 | 1 |  |
| Botiidae_Sinibotia_superciliaris | 1 | 0 | 1 |  |
| Nemacheilidae_Homatula_potanini | 1 | 0 | 1 | Nichols 1925. |
| Cobitidae_Misgurnus_anguillicaudatus | 1 | 0 | 1 | Luo et al. 2016 |
| Danionidae_Danio_rerio | 1 | 0 | 0 |  |
| Tincidae_Tinca_tinca | 1 | 0 | 0 |  |
| Leuciscidae_Pimephales_promelas | 1 | 0 | 0 |  |
| Leuciscidae_Leuciscus_waleckii | 1 | 0 | 0 |  |
| Cyprinidae_Cyprinus_carpio | 1 | 0 | 0 |  |
| Cyprinidae_Sinocyclocheilus_rhinocerous | 1 | 0 | 0 |  |
| Cyprinidae_Sinocyclocheilus_anshuiensis | 1 | 0 | 0 |  |
| Cyprinidae_Sinocyclocheilus_grahami | 1 | 0 | 0 |  |
| Distichodontidae_Distichodus_sexfasciatus | 1 | 0 | 0 |  |
| Serrasalmidae_Pygocentrus_nattereri | 1 | 0 | 0 |  |
| Erythrinidae_Erythrinus_erythrinus | 1 | 0 | 0 |  |
| Hepsetidae_Hepsetus_odoe | 1 | 0 | 0 |  |
| Gasteropelecidae_Gasteropelecus_sp | 1 | 0 | 0 |  |
| Characidae_Astyanax_mexicanus | 1 | 0 | 0 |  |
| Characidae_Thayeria_boehlkei | 1 | 0 | 0 |  |
| Characidae_Hemigrammus_bleheri | 1 | 0 | 0 |  |
| Apteronotidae_Apteronotus_albifrons | 1 | 0 | 1 |  |
| Gymnotidae_Electrophorus_electricus | 0 | 0 | 0 |  |
| Rhamphichthyidae_Rhamphichthys_rostratus | 1 | 0 | 1 |  |
| Callichthyidae_Corydoras_julii | 0 | 1 | 1 | Liu et a. 2016 |
| Loricariidae_Pterygoplichthys_pardalis | 0 | 1 | 1 |  |
| Plotosidae_Plotosus_lineatus | 0 | 0 | 1 |  |
| Pangasiidae_Pangasianodon_hypophthalamus | 0 | 0 | 1 |  |
| Siluridae_Silurus_asotus | 0 | 0 | 1 |  |
| Ictaluridae_Ictalurus_punctatus | 0 | 0 | 1 |  |
| Bagridae_Hemibagrus_guttatus | 0 | 0 | 1 |  |
| Sisoridae_Glyptothorax_sinensis | 0 | 0 | 1 |  |
| Amblycipitidae_Liobagrus_styani | 0 | 0 | 1 |  |
| Lepidogalaxiidae_Lepidogalaxias_salamandroides | 1 | 0 | 0 |  |
| Esocidae_Esox_lucius | 1 | 0 | 0 |  |
| Umbridae_Umbra_pygmae | 1 | 0 | 0 |  |
| Salmonidae_Coregonus_clupeaformis | 1 | 0 | 0 |  |
| Salmonidae_Thymallus_thymallus | 1 | 0 | 0 |  |
| Salmonidae_Salmo_salar | 1 | 0 | 0 |  |
| Salmonidae_Oncorhynchus_mykiss | 1 | 0 | 0 |  |
| Salmonidae_Salvelinus_fontinalis | 1 | 0 | 0 |  |
| Argentinidae_Argentina_sp | 1 | 0 | 0 |  |
| Galaxiidae_Galaxias_maculatus | 0 | 0 | 0 | Waters et al. 2000 |
| Galaxiidae_Galaxiella_nigrostriata | 0 | 0 | 0 |  |
| Stomiidae_Borostomias_antarcticus | 1 | 0 | 0 |  |
| Osmeridae_Osmerus_eperlanus | 1 | 0 | 0 |  |
| Plecoglossidae_Plecoglossus_altivelis | 1 | 0 | 0 |  |
| Salangidae_Protosalanx_hyalocranius | 1 | 0 | 0 |  |
| Synodontidae_Synodus_intermedius | 1 | 0 | 0 |  |
| Chlorophthalmidae_Parasudis_fraserbrunneri | 1 | 0 | 0 |  |
| Chlorophthalmidae_Chlorophthalmus_agassizi | 1 | 0 | 0 |  |
| Ateleopodidae_Guentherus_altivela | 1 | 0 | 0 |  |
| Myctophidae_Benthosema_glaciale | 1 | 0 | 0 |  |
| Polymixiidae_Polymixia_japonica | 1 | 0 | 0 |  |
| Amblyopsidae_Typhlichthys_subterraneus | 1 | 0 | 0 |  |
| Percopsidae_Percopsis_transmontana | 1 | 0 | 0 |  |
| Percopsidae_Percopsis_omiscomaycus | 1 | 0 | 0 |  |
| Zeidae_Zeus_faber | 1 | 0 | 0 |  |
| Parazenidae_Cyttopsis_roseus | 1 | 0 | 0 |  |
| Parazenidae_Cyttopsis_sp | 1 | 0 | 0 |  |
| Stylephoridae_Stylephorus_chordatus | 1 | 0 | 0 |  |
| Bregmacerotidae_Bregmaceros_cantori | 1 | 0 | 0 |  |
| Merlucciidae_Merluccius_polli | 1 | 0 | 0 |  |
| Merlucciidae_Merluccius_capensis | 1 | 0 | 0 |  |
| Merlucciidae_Merluccius_merluccius | 1 | 0 | 0 |  |
| Melanonidae_Melanorus_zugmayeri | 1 | 0 | 0 |  |
| Muraenolepididae_Muraenolepis_marmoratus | 1 | 0 | 0 |  |
| Trachyrincidae_Trachyrincus_scabrus | 1 | 0 | 1 |  |
| Trachyrincidae_Trachyrincus_murrayi | 1 | 0 | 1 |  |
| Moridae_Mora_moro | 1 | 0 | 0 |  |
| Moridae_Laemonema_laureysi | 1 | 0 | 0 |  |
| Bathygadidae_Bathygadus_melanobranchus | 1 | 0 | 1 |  |
| Macrouridae_Macrourus_berglax | 1 | 0 | 1 |  |
| Macrouridae_Malacocephalus_occidentalis | 1 | 0 | 1 |  |
| Phycidae_Phycis_phycis | 1 | 0 | 0 |  |
| Phycidae_Phycis_blennoides | 1 | 0 | 0 |  |
| Lotidae_Lota_lota | 1 | 0 | 0 |  |
| Lotidae_Molva_molva | 1 | 0 | 0 |  |
| Lotidae_Brosme_brosme | 1 | 0 | 0 |  |
| Gadidae_Trisopterus_minutus | 1 | 0 | 0 |  |
| Gadidae_Gadiculus_argenteus | 1 | 0 | 0 |  |
| Gadidae_Pollachius_virens | 1 | 0 | 0 |  |
| Gadidae_Merlangius_merlangus | 1 | 0 | 0 |  |
| Gadidae_Melanogrammus_aeglefinus | 1 | 0 | 0 |  |
| Gadidae_Arctogadus_glacialis | 1 | 0 | 0 |  |
| Gadidae_Boreogadus_saida | 1 | 0 | 0 |  |
| Gadidae_Theragra_chalcogramma | 1 | 0 | 0 |  |
| Gadidae_Gadus_morhua | 1 | 0 | 0 |  |
| Regalecidae_Regalecus_glesne | 0 | 0 | 0 | Ruiz et al. 2010 |
| Lampridae_Lampris_guttatus | 1 | 0 | 0 |  |
| Monocentridae_Monocentris_japonica | 1 | 0 | 0 |  |
| Berycidae_Beryx_splendens | 1 | 0 | 0 |  |
| Stephanoberycidae_Acanthochaenus_luetkenii | 1 | 0 | 0 |  |
| Rondeletiidae_Rondeletia_loricata | 1 | 0 | 0 |  |
| Holocentridae_Myripristis_berndti | 1 | 0 | 0 |  |
| Holocentridae_Myripristis_jacobus | 1 | 0 | 0 |  |
| Holocentridae_Sargocentron_rubrum | 1 | 0 | 0 |  |
| Holocentridae_Holocentrus_rufus | 1 | 0 | 0 |  |
| Holocentridae_Neoniphon_sammara | 1 | 0 | 0 |  |
| Holocentridae_Neoniphon_vexillarium | 1 | 0 | 0 |  |
| Ophidiidae_Brotula_barbata | 1 | 0 | 1 |  |
| Ophidiidae_Carapus_acus | 0 | 0 | 1 | Ohashi et al. 2018 |
| Ophidiidae_Lamprogrammus_exutus | 1 | 0 | 1 |  |
| Batrachoididae_Chatrabus_melanurus | 0 | 0 | 1 | Greenfield et al. 2008 |
| Batrachoididae_Porichthys_notatus | 0 | 0 | 1 |  |
| Batrachoididae_Batrachomoeus_trispinosus | 0 | 0 | 1 |  |
| Apogonidae_Apogonichthyoides_cathetogramma | 1 | 0 | 0 |  |
| Apogonidae_Phaeoptyx_conklini | 1 | 0 | 0 |  |
| Gobiidae_Periophthalmodon_schlosseri | 1 | 0 | 1 |  |
| Gobiidae_Periophthalmus_magnuspinnatus | 1 | 0 | 1 |  |
| Gobiidae_Scartelaos_histophorus | 1 | 0 | 1 |  |
| Gobiidae_Boleophthalmus_pectinirostris | 1 | 0 | 1 |  |
| Gobiidae_Coryphopterus_lipernes | 1 | 0 | 1 |  |
| Gobiidae_Lesueurigobius_cf_sanzoi | 1 | 0 | 1 |  |
| Gobiidae_Glossogobius_aureus | 1 | 0 | 1 |  |
| Gobiidae_Exyrias_puntang | 1 | 0 | 1 |  |
| Gobiidae_Istigobius_decoratus | 1 | 0 | 1 |  |
| Nomeidae_Nomeus_gronovii | 1 | 0 | 0 |  |
| Stromateidae_Pampus_argenteus | 1 | 0 | 0 |  |
| Scombridae_Scomberomorus_regalis | 1 | 0 | 0 |  |
| Scombridae_Scomber_scombrus | 1 | 0 | 0 |  |
| Scombridae_Thunnus_albacares | 1 | 0 | 0 |  |
| Scombridae_Thunnus_orientalis | 1 | 0 | 0 |  |
| Aulostomidae_Aulostomus_maculatus | 1 | 0 | 0 |  |
| Aulostomidae_Aulostomus_sp | 1 | 0 | 0 |  |
| Mullidae_Parupeneus_indicus | 1 | 0 | 1 |  |
| Callionymidae_Foetorepus_agassizii | 0 | 0 | 1 | Sadovy et al. 2005 |
| Syngnathidae_Syngnathoides_biaculeatus | 0 | 1 | 1 | Lourie et al. 2016 |
| Syngnathidae_Syngnathus_scovelli | 0 | 1 | 1 |  |
| Syngnathidae_Hippocampus_erectus | 0 | 1 | 1 |  |
| Percidae_Perca_fluviatilis | 1 | 0 | 0 |  |
| Percophidae_Chrionema_squamentum | 1 | 0 | 1 |  |
| Serranidae_Pronotogrammus_martinicensis | 1 | 0 | 1 |  |
| Nototheniidae_Lepidonotothen_nudifrons | 1 | 0 | 1 |  |
| Nototheniidae_Notothenia_coriiceps | 1 | 0 | 1 |  |
| Bathydraconidae_Gymnodraco_acuticeps | 1 | 0 | 1 |  |
| Channichthydae_Chaenocephalus_aceratus | 1 | 0 | 1 |  |
| Peristediidae_Peristedion_brevirostre | 1 | 0 | 1 |  |
| Anoplopomatidae_Anoplopoma_fimbria | 1 | 0 | 1 |  |
| Gasterosteidae_Gasterosteus_aculeatus | 0 | 1 | 1 | Hebig et al. 1978 |
| Cyclopteridae_Cyclopterus_lumpus | 0 | 1 | 0 | Märss et al 2010 |
| Cottidae_Cottus_rhenanus | 0 | 1 | 1 | Cheng et al. 2016 |
| Cottidae_Myoxocephalus_scorpius | 0 | 1 | 1 |  |
| Scorpaenidae_Scorpaenopsis_cirrosa | 1 | 0 | 1 |  |
| Synanceiidae_Synanceia_verrucosa | 1 | 0 | 1 |  |
| Scorpaenidae_Dendrochirus_zebra | 1 | 0 | 1 |  |
| Scorpaenidae_Pontinus_castor | 1 | 0 | 1 |  |
| Sebastidae_Sebastes_norvegicus | 1 | 0 | 0 |  |
| Sebastidae_Sebastes_rubrivinctus | 1 | 0 | 0 |  |
| Sebastidae_Sebastes_nigrocinctus | 1 | 0 | 0 |  |
| Epigonidae_Epigonus_sp | 1 | 0 | 0 |  |
| Lateolabracidae_Lateolabrax_maculatus | 1 | 0 | 0 |  |
| Gerreidae_Gerres_filamentosus | 1 | 0 | 0 |  |
| Gerreidae_Eucinostomus_jonesi | 1 | 0 | 0 |  |
| Pinguipedidae_Parapercis_xanthozona | 1 | 0 | 0 |  |
| Labridae_Thalassoma_bifasciatum | 1 | 0 | 0 |  |
| Scaridae_Scarus_iseri | 1 | 0 | 0 |  |
| Scaridae_Scarus_ghobban | 1 | 0 | 0 |  |
| Labridae_Symphodus_melops | 1 | 0 | 0 |  |
| Labridae_Labrus_bergylta | 1 | 0 | 0 |  |
| Terapontidae_Terapon_jarbua | 1 | 0 | 0 |  |
| Oplegnathidae_Oplegnathus_punctatus | 1 | 0 | 0 |  |
| Cirrhitidae_Amblycirrhitus_pinos | 1 | 0 | 0 |  |
| Centrarchidae_Micropterus_floridanus | 1 | 0 | 0 |  |
| Percichthyidae_Siniperca_scherzeri | 1 | 0 | 0 |  |
| Percichthyidae_Coreoperca_whiteheadi | 1 | 0 | 0 |  |
| Moronidae_Morone_saxatilis | 1 | 0 | 0 |  |
| Moronidae_Dicentrarchus_labrax | 1 | 0 | 0 |  |
| Ephippidae_Chaetodipterus_faber | 1 | 0 | 0 |  |
| Drepaneidae_Drepane_punctata | 1 | 0 | 0 |  |
| Datnioididae_Datnioides_microlepis | 1 | 0 | 0 |  |
| Lutjanidae_Lutjanus_sebae | 1 | 0 | 0 |  |
| Lutjanidae_Lutjanus_fulviflamma | 1 | 0 | 0 |  |
| Haemulidae_Haemulon_flavolineatum | 1 | 0 | 0 |  |
| Haemulidae_Haemulon_chrysargyreum | 1 | 0 | 0 |  |
| Sciaenidae_Equetus_punctatus | 1 | 0 | 0 |  |
| Sciaenidae_Larimichthys_crocea | 1 | 0 | 0 |  |
| Sciaenidae_Miichthys_miiuy | 1 | 0 | 0 |  |
| Acanthuridae_Acanthurus_tractus | 1 | 0 | 0 |  |
| Pomacanthidae_Pomacanthus_paru | 1 | 0 | 0 |  |
| Leiognathidae_Photopectoralis_bindus | 1 | 0 | 0 |  |
| Chaetodontidae_Chaetodon_auriga | 1 | 0 | 0 |  |
| Siganidae_Siganus_guttatus | 1 | 0 | 0 |  |
| Sparidae_Evynnis_cardinalis | 1 | 0 | 0 |  |
| Sparidae_Spondyliosoma_cantharus | 1 | 0 | 0 |  |
| Sparidae_Acanthopagrus_latus | 1 | 0 | 0 |  |
| Sparidae_Acanthopagrus_schlegelii | 1 | 0 | 0 |  |
| Caproidae_Antigonia_capros | 1 | 0 | 0 |  |
| Priacanthidae_Priacanthus_tayenus | 1 | 0 | 0 |  |
| Chaunacidae_Chaunax_pictus | 1 | 0 | 0 |  |
| Antennariidae_Antennarius_striatus | 1 | 0 | 0 |  |
| Ostraciidae_Lactoria_cornuta | 1 | 0 | 0 |  |
| Ostraciidae_Ostracion_rhinorhynchos | 1 | 0 | 0 |  |
| Balistidae_Pseudobalistes_fuscus | 1 | 0 | 0 |  |
| Molidae_Mola_mola | 1 | 0 | 0 |  |
| Diodontidae_Diodon_holocanthus | 1 | 0 | 0 |  |
| Tetraodontidae_Tetraodon_nigroviridis | 1 | 0 | 0 |  |
| Tetraodontidae_Takifugu_flavidus | 1 | 0 | 0 |  |
| Tetraodontidae_Takifugu_rubripes | 1 | 0 | 0 |  |
| Synbranchidae_Monopterus_albus | 0 | 0 | 1 | Liem et al. 1967 |
| Mastacembelidae_Macrognathus_aculeatus | 1 | 0 | 1 |  |
| Mastacembelidae_Mastacembelus_armatus | 1 | 0 | 1 |  |
| Channidae_Channa_micropeltes | 1 | 0 | 0 |  |
| Channidae_Channa_argus | 1 | 0 | 0 |  |
| Channidae_Channa_gachua | 1 | 0 | 0 |  |
| Osphronemidae_Osphronemus_goramy | 1 | 0 | 0 |  |
| Helostomatidae_Helostoma_temminckii | 1 | 0 | 0 |  |
| Anabantidae_Anabas_testudineus | 1 | 0 | 0 |  |
| Centropomidae_Centropomus_undecimalis | 1 | 0 | 0 |  |
| Centropomidae_Lates_calcarifer | 1 | 0 | 0 |  |
| Toxotidae_Toxotes_jaculatrix | 1 | 0 | 0 |  |
| Coryphaenidae_Coryphaena_hippurus | 1 | 0 | 0 |  |
| Carangidae_Trachinotus_ovatus | 1 | 0 | 0 |  |
| Carangidae_Seriola_lalandi | 1 | 0 | 0 |  |
| Carangidae_Selene_dorsalis | 1 | 0 | 0 |  |
| Carangidae_Caranx_ignobilis | 1 | 0 | 0 |  |
| Polynemidae_Polynemus_dubius | 1 | 0 | 0 |  |
| Soleidae_Solea_ovata | 1 | 0 | 1 |  |
| Cynoglossidae_Cynoglossus_semilaevis | 1 | 0 | 1 |  |
| Scophthalmidae_Scophthalmus_maximus | 1 | 0 | 1 |  |
| Paralichthydae_Paralichthys_olivaceus | 1 | 0 | 1 |  |
| Pleuronectidae_Hippoglossus_hippoglossus | 1 | 0 | 1 |  |
| Pleuronectidae_Pseudopleuronectes_yokohamae | 1 | 0 | 1 |  |
| Ambassidae_Parambassis_pulcinella | 1 | 0 | 0 |  |
| Mugilidae_Liza_haematocheila | 1 | 0 | 0 |  |
| Mugilidae_Mugil_cephalus | 1 | 0 | 0 |  |
| Pseudochromidae_Pseudochromis_fuscus | 1 | 0 | 0 |  |
| Pomacentridae_Stegastes_partitus | 1 | 0 | 0 |  |
| Pomacentridae_Amphiprion_melanopus | 1 | 0 | 0 |  |
| Pomacentridae_Dascyllus_trimaculatus | 1 | 0 | 0 |  |
| Pomacentridae_Chromis_chromis | 1 | 0 | 0 |  |
| Grammatidae_Lipogramma_evides | 1 | 0 | 0 |  |
| Opistognathidae_Opistognathus_aurifrons | 1 | 0 | 0 |  |
| Gobiesocidae_Tomicodon_sp | 0 | 0 | 1 | Yamada et al. 2009 |
| Gobiesocidae_Acyrtus_rubiginosus | 0 | 0 | 1 |  |
| Tripterygiidae_Enneanectes_sp | 1 | 0 | 1 |  |
| Blenniidae_Parablennius_parvicornis | 0 | 0 | 1 | Hundt et al. 2014 |
| Chaenopsidae_Acanthemblemaria_spinosa | 0 | 0 | 1 |  |
| Cichlidae_Parachromis_managuensis | 1 | 0 | 0 |  |
| Cichlidae_Amphilophus_citrinellus | 1 | 0 | 0 |  |
| Cichlidae_Oreochromis_niloticus | 1 | 0 | 0 |  |
| Cichlidae_Neolamprologus_brichardi | 1 | 0 | 0 |  |
| Cichlidae_Haplochromis_burtoni | 1 | 0 | 0 |  |
| Cichlidae_Pundamilia_nyererei | 1 | 0 | 0 |  |
| Cichlidae_Maylandia_zebra | 1 | 0 | 0 |  |
| Cichlidae_Mchenga_conophoros | 1 | 0 | 0 |  |
| Cichlidae_Labeotropheus_fuelleborni | 1 | 0 | 0 |  |
| Cichlidae_Rhamphochromis_esox | 1 | 0 | 0 |  |
| Cichlidae_Melanochromis_auratus | 1 | 0 | 0 |  |
| Hemiramphidae_Hyporhamphus_intermedius | 1 | 0 | 0 |  |
| Adrianichthyidae_Oryzias_mekongensis | 1 | 0 | 0 |  |
| Adrianichthyidae_Oryzias_latipes | 1 | 0 | 0 |  |
| Pseudomugilidae_Pseudomugil_paskai | 1 | 0 | 0 |  |
| Melanotaeniidae_Glossolepis_incisus | 1 | 0 | 0 |  |
| Melanotaeniidae_Melanotaenia_praecox | 1 | 0 | 0 |  |
| Atherinopsidae_Menidia_menidia | 1 | 0 | 0 |  |
| Atherinopsidae_Basilichthys_microlepidotus | 1 | 0 | 0 |  |
| Atherinopsidae_Odontesthes_bonariensis | 1 | 0 | 0 |  |
| Aplocheilidae_Pachypanchax_sakaramyi | 1 | 0 | 0 |  |
| Nothobranchiidae_Nothobranchius_furzeri | 1 | 0 | 0 |  |
| Aplocheilidae_Aplocheilus_lineatus | 1 | 0 | 0 |  |
| Rivulidae_Austrofundulus_limnaeus | 1 | 0 | 0 |  |
| Rivulidae_Kryptolebias_marmoratus | 1 | 0 | 0 |  |
| Goodeidae_Ameca_splendens | 1 | 0 | 0 |  |
| Fundulidae_Fundulus_heteroclitus | 1 | 0 | 0 |  |
| Cyprinodontidae_Cyprinodon_variegatus | 1 | 0 | 0 |  |
| Cyprinodontidae_Cyprinodon_nevadensis_pectoralis | 1 | 0 | 0 |  |
| Poeciliidae_Poecilia_reticulata | 1 | 0 | 0 |  |
| Poeciliidae_Poecilia_formosa | 1 | 0 | 0 |  |
| Poeciliidae_Poeciliopsis_prolifica | 1 | 0 | 0 |  |
| Poeciliidae_Gambusia_affinis_whole | 1 | 0 | 0 |  |
| Poeciliidae_Xiphophorus_hellerii | 1 | 0 | 0 |  |
| Poeciliidae_Xiphophorus_maculatus | 1 | 0 | 0 |  |
| Poeciliidae_Xiphophorus_couchianus | 1 | 0 | 0 |  |

**Table S2** List of 11,638 species of Actinopterygii from the dataset in Rabosky et al. (2018) and their classification for three characters. Presence (1) absence (0) of scales, of trunk bony plates (TBP), and finally the habitat preference (0 = open water, 1 = benthic). The information about the traits was collected in two books (Moyle and Cech 2004; Nelson et al. 2016) and in Fishbase (Froese and Pauly 2011). When information was lacking or unclear in these three main sources, species characteristics were extracted from the specialized literature, given in the column "Reference".

***File attached (due to its large size)***

**Table S3** Model selection for the two datasets we used, the 304 species of Hughes *et al.* (2018) and the 11,638 species of Rabosky *et al.* (2018) and for two different binary traits, presence / absence of scales and presence / absence of trunk bony plates (TBP).

|  | 304 species dataset | | 11,638 species dataset | |
| --- | --- | --- | --- | --- |
|  | Scales | TBP | Scales | TBP |
| AIC (ER) | 126.5965 | 51.55692 | 889.6491 | 550.04330 |
| AIC(ARD) | 128.4000 | 53.31422 | 891.49015 | 514.35020 |
|  |  |  |  |  |
| Uniform | -71.814131 | -32.107809 | -458.633667 | -263.965343 |
| Exponential | -68.993721 | -28.606158 | -456.057166 | -260.061577 |
| LogBF | 2*(-68.993721-(-71.814131))=  5,640818 | 2*(-28.606158-(-32.107809))=  7,012302 | 2*(-456.057166 - -458.633667)= 5.153002 | 2*(-260.061577 - - -263.965343)= 7.807532 |

**Table S4**. Number of scaleless species living in open waters, which are included in the two datasets we analyzed: Hughes *et al.* (2018) and Rabosky *et al.* (2018).

| Hughes et al. 2018 dataset | Rabosky et al. 2018 dataset | Family | Species (Fishbase) |
| --- | --- | --- | --- |
| 2 | 47 | Galaxiidae | 53 |
| 0 | 41 | Stomiidae | 287 |
| 0 | 17 | Salangidae | 20 |
| 0 | 12 | Trichomyteridae | 288 |
| 0 | 6 | Cyprinidae | 3160 |
| 0 | 4 | Schibleidae | 46 |
| 0 | 4 | Serrivomeridae | 9 |
| 0 | 3 | Evermanellidae | 8 |
| 0 | 3 | Nemichthyidae | 9 |
| 1 | 2 | Regalecidae | 3 |
| 0 | 2 | Paralepididae | 58 |
| 0 | 2 | Alepisauridae | 2 |
| 0 | 2 | Saccopharyngidae | 10 |
| 0 | 2 | Giganturidae | 2 |
| 0 | 1 | Monocentridae | 4 |
| 0 | 1 | Omosudidae | 1 |
| 0 | 1 | Synaphobranchidae | 39 |
| 1 | 1 | Cyclopteridae | 27 |
| 0 | 1 | Alepocephalidae | 98 |

**References**

Arnold, R.J., and Pietsch, T. W. 2012. Evolutionary history of frogfishes (Teleostei: Lophiiformes: Antennariidae): A molecular approach. Molecular Phylogenetics and Evolution, 62(1), 117–129. https://doi.org/10.1016/j.ympev.2011.09.012

Böhlke, E.B., Böhlke J., Leiby, M., McCoscer, J.E., Bertelsen, E., Robinson, C., Robins, C.R., Smith, D.G., and Tighe, K.A. 1989. Orders Anguilliformes and Saccopharyngiformes: Part 9, Volume 1. Yale University Press.

Cheng, J., Sedlazek, F., Altmüller, J., and Nolte, A. W. 2015. Ectodysplasin signalling genes and phenotypic evolution in sculpins (*Cottus*). *Proceedings of the Royal Society B: Biological Sciences*, 282(1815). https://doi.org/10.1098/rspb.2015.0746

Froese, R. and Pauly., D. 2011. Fishbase. www.fishbase.org

Greenfield, D. W., Winterbottom, R., and Collette, B. B. 2008. Review of the toadfish genera (Teleostei: Batrachoididae). *Proceedings of the California Academy of Sciences*, 59(14–20), 665–710.

Hebig, W., and Wootton, R.J. 1978. The Biology of the Sticklebacks. – Mit 77 Abb., X/387 S. London, New York, San Francisco: Aca emic Press, doi:10.1002/iroh.19780630314

Hilton, E. J., Grande, L., and Bemis, W. E. 2011. Skeletal Anatomy of the Shortnose Sturgeon, Acipenser brevirostrum Lesueur, 1818, and the Systematics of Sturgeons (Acipenseriformes, Acipenseridae). *Fieldiana Life and Earth Sciences*, 3, 1–168. https://doi.org/10.3158/2158-5520-3.1.1

Hughes, L.C., Ortí, G., Huang, Y., Sun, Y., Baldwin, C.C., Thompson, A.W., et al. 2018. Comprehensive phylogeny of ray-finned fishes (Actinopterygii) based on transcriptomic and genomic data. *Proceedings of the National Academy of Sciences*. U. S. A. 115: 6249–6254.

Hundt, P. J., Iglésias, S. P., Hoey, A. S., and Simons, A. M. 2014. A multilocus molecular phylogeny of combtooth blennies (Percomorpha: Blennioidei: Blenniidae): Multiple invasions of intertidal habitats. *Molecular Phylogenetics and Evolution*, 70(1), 47–56. https://doi.org/10.1016/j.ympev.2013.09.001

Johnson, D.G., Ida, H., Sakaue, J., Sado, T., Asahida, T., and Miya, M. 2012. A “living fossil” eel (Anguilliformes: Protanguillidae, fam. nov.) from an undersea cave in Palau. *Proceedings of the Royal Society B: Biological Sciences*, 279(1730), 934–943. https://doi.org/10.1098/rspb.2011.1289

Kenaley, C.P., Devaney, S. C., and Fjeran, T.T. 2014. The complex evolutionary history of seeing red: Molecular phylogeny and the evolution of an adaptive visual system in deep-sea dragonfishes (Stomiiformes: Stomiidae). *Evolution*, 68(4), 996–1013. https://doi.org/10.1111/evo.12322

Liem, K. F. 1967. Functional Morphology of the integumentary, respiratory , and digestive systems of the synbranchoid fish *Monopterus albus*. *American Society of Ichthyologists and Herpetologists*, 1967(2), 375–388.

Liu, Z., Liu, S., Yao, J., Bao, L., Zhang, J., Li, Y., and Waldbieser, G. C. 2016. The channel catfish genome sequence provides insights into the evolution of scale formation in teleosts. *Nature Communications*, 7, 11757. https://doi.org/10.1038/ncomms11757

Lourie, S. A., Pollom, R. A., and Foster, S. J. 2016. A global revision of the seahorses *Hippocampus* Rafinesque 1810 (Actinopterygii: Syngnathiformes): Taxonomy and biogeography with recommendations for further research. *Zootaxa*, 4146 (1), 001–066. https://doi.org/10.11646/zootaxa.4146.1.1

Luo, W. W., Liu, C. S., Cao, X. J., Huang, L. F., and Huang, S. Q. 2016. Precision of age estimations from scales, otoliths, vertebrae, opercular bones and cleithra of two loaches, *Misgurnus anguillicaudatus* and *Paramisgurnus dabryanus*. *Folia Zoologica*, 65(3), 183–188. https://doi.org/10.25225/fozo.v65.i3.a2.2016

Märss, T., Lees, J., Wilson, M. V. H., Saat, T., and Špilev, H. 2010. The morphology and sculpture of ossicles in the Cyclopteridae and Liparidae (Teleostei) of the Baltic Sea. *Estonian Journal of Earth Sciences*, 59(4), 263–276. https://doi.org/10.3176/earth.2010.4.03

Moyle, P.B. and Cech, J.J. 2004. Fishes, An introduction to Ichthyology, 5th ed. (B. Cummings, ed).

Near, T.J., MacGuigan, D. J., Parker, E., Struthers, C.D., Jones, C.D., and Dornburg, A. 2018. Phylogenetic analysis of Antarctic notothenioids illuminates the utility of RADseq for resolving Cenozoic adaptive radiations. *Molecular Phylogenetics and Evolution*, 129, 268–279. https://doi.org/10.1016/j.ympev.2018.09.001

Nelson, J.S., Grande, T.C. and Wilson, M.V.H. 2016. Fishes of the World, 5th ed. Wiley.

Nichols, J. T. 1925. *Nemacheilus* and related loaches in China. *American Museum Novitates*, 171.

Ohashi, S. 2018. Morphology of a unique ophidiid, *Hypopleuron caninum* Radcliffe 1913 (Ophidiiformes, Ophidiidae, Neobythitinae), suggesting a close relationship with the family Carapidae. *Zootaxa*, 4521(4), 499–538. https://doi.org/10.11646/zootaxa.4521.4.2

Rabosky, D. L., Chang, J., Title, P.O., Cowman, P.F., Sallan, L., Friedman, M., Kaschner, K., Garilao, C., Near, T. J., Coll, M., and Alfaro, M. E. 2018. An inverse latitudinal gradient in speciation rate for marine fishes. *Nature*, 559(7714), 392–395. https://doi.org/10.1038/s41586-018-0273-1

Radchenko, O.A., Chereshnev, I. A., Petrovskaya, A. V., and Antonenko, D. V. 2011. Relationships and position of wrymouths of the family Cryptacanthodidae in the system of the suborder Zoarcoidei (Pisces, Perciformes). *Journal of Ichthyology*, 51(7), 487–499. https://doi.org/10.1134/S0032945211040163

Rice, A.N., and Bass, A. H. 2009. Novel vocal repertoire and paired swimbladders of the three-spined toadfish, *Batrachomoeus trispinosus*: Insights into the diversity of the Batrachoididae. *Journal of Experimental Biology*, 212(9), 1377–1391. https://doi.org/10.1242/jeb.028506

Ruiz, A. E., and Gosztonyi, A. E. 2010. Records of regalecid fishes in Argentine waters. *Zootaxa*, 66(2509), 62–66. https://doi.org/10.11646/zootaxa.2509.1.5

Sadovy, Y., Randall, J. E., and Rasotto, M. B. 2005. Skin structure in six dragonet species (Gobiesociformes; Callionymidae): Interspecific differences in glandular cell types and mucus secretion. *Journal of Fish Biology*, 66(5), 1411–1418. https://doi.org/10.1111/j.0022-1112.2005.00692.x

Santini, F., Kong, X., Sorenson, L., Carnevale, G., Mehta, R. S., and Alfaro, M. E. 2013. A multi-locus molecular timescale for the origin and diversification of eels (Order: Anguilliformes). *Molecular Phylogenetics and Evolution*, 69(3), 884–894. https://doi.org/10.1016/j.ympev.2013.06.016

Smith, W.L., Everman, E., and Richardson, C. 2018. Phylogeny and taxonomy of flatheads, scorpionfishes, sea robins, and stonefishes (Percomorpha: Scorpaeniformes) and the Evolution of the lachrymal saber. *Copeia*, 106(1), 94–119. https://doi.org/10.1643/CG-17-669

Turanov, S.V., Kartavtsev, Y.P., Lee, Y. H., and Jeong, D. 2017. Molecular phylogenetic reconstruction and taxonomic investigation of eelpouts (Cottoidei: Zoarcales) based on Co-1 and Cyt-b mitochondrial genes. *Mitochondrial DNA Part A: DNA Mapping, Sequencing, and Analysis*, 28(4), 547–557. https://doi.org/10.3109/24701394.2016.1155117

Waters, J. M., López, J. A., and Wallis, G. P. 2000. Molecular phylogenetics and biogeography of galaxiid fishes (Osteichthyes: Galaxiidae): Dispersal, vicariance, and the position of *Lepidogalaxias salamandroides*. *Systematic Biology*, 49(4), 777–795. https://doi.org/10.1080/106351500750049824

Weisel, G. F. 1975. The integument of the paddlefish, *Polyodon spathula*. *Journal of Morphology*, 145(2), 143–150. https://doi.org/10.1002/jmor.1051450203

Yamada, T., Sugiyama, T., Tamaki, N., Kawakita, A., and Kato, M. 2009. Adaptive radiation of gobies in the interstitial habitats of gravel beaches accompanied by body elongation and excessive vertebral segmentation. *BMC Evolutionary Biology*, 9(1), 1–14. https://doi.org/10.1186/1471-2148-9-145
