# Supplementary figures and images for: From scales to armor: Scale losses and trunk bony plate gains in ray‐finned fishes
Source: Evol Lett. 2021 Mar 23;5(3):240–50. doi: 10.1002/evl3.219 (PMC8190451; doi:10.1002/evl3.219)

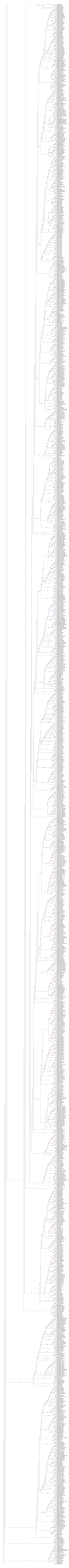

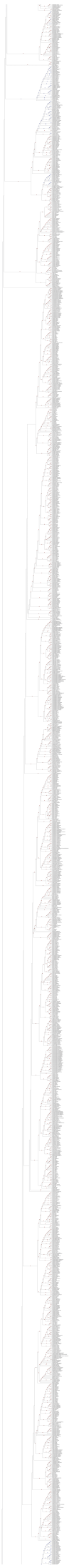

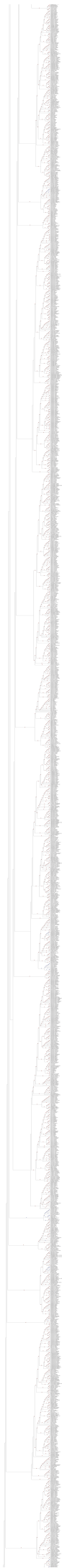

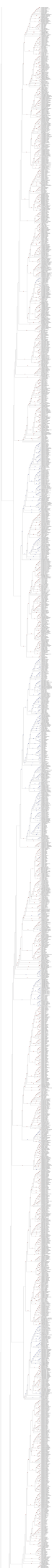

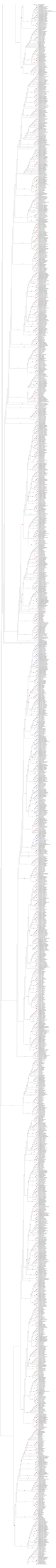

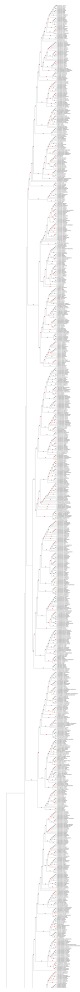

Supplement: Supplementary file 3 — Supplementary Materials [file EVL3-5-240-s004.zip › S5_Bayestrait_Scales_Rabosky_etal_data.pdf]

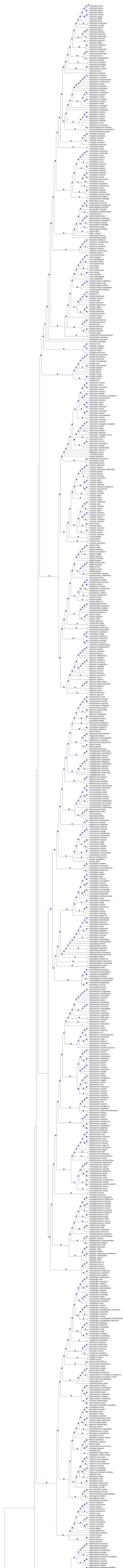

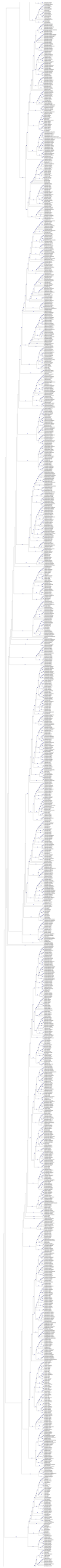

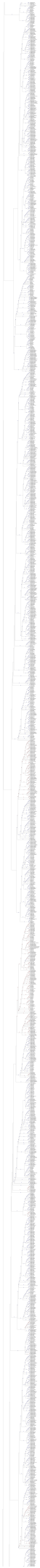

Supplement: Supplementary file 4 — Supplementary Materials [file EVL3-5-240-s002.zip › S10_Bayestrait_TBP_Rabosky_etal_data.pdf]
